# Stevens–Johnson Syndrome and Toxic Epidermal Necrolysis: Analysis of the Russian Database of Spontaneous Reports

**DOI:** 10.3390/ph17060675

**Published:** 2024-05-24

**Authors:** Sergey Zyryanov, Irina Asetskaya, Olga Butranova, Elizaveta Terekhina, Vitaly Polivanov, Alexander Yudin, Kristina Samsonova

**Affiliations:** 1 Department of General and Clinical Pharmacology, Peoples’ Friendship University of Russia Named after Patrice Lumumba (RUDN), 6 Miklukho-Maklaya St., 117198 Moscow, Russia; zyryanov-sk@rudn.ru (S.Z.); asetskaya-il@rudn.ru (I.A.); 1032173831@rudn.ru (E.T.); 1042210102@rudn.university (K.S.); 2 Moscow City Health Department, City Clinical Hospital No. 24, State Budgetary Institution of Healthcare of the City of Moscow, Pistzovaya Str. 10, 127015 Moscow, Russia; youdine@gmail.com; 3 Pharmacovigilance Center, Information and Methodological Center for Expert Evaluation, Record and Analysis of Circulation of Medical Products under the Federal Service for Surveillance in Healthcare, 4-1 Slavyanskaya Square, 109074 Moscow, Russia; pvit74@gmail.com; 4 Russian National Research Medical University Named after N.I. Pirogov, St. Ostrovityanova, 1, 117997 Moscow, Russia

**Keywords:** pharmacovigilance, severe cutaneous adverse drug reactions, drug-induced allergy, anti-infectives for systemic use, antiepileptics, lamotrigine

## Abstract

(1) Background: Stevens–Johnson syndrome (SJS) and toxic epidermal necrolysis (TEN) are extremely severe cutaneous adverse drug reactions which are relatively rare in routine clinical practice. An analysis of a national pharmacovigilance database may be the most effective method of obtaining information on SJS and TEN. (2) Methods: Design—a retrospective descriptive pharmacoepidemiologic study of spontaneous reports (SRs) with data on SJS and TEN retrieved from the Russian National Pharmacovigilance database for the period from 1 April 2019 to 31 December 2023. Descriptive statistics was used to assess the demographic data of patients and the structure of suspected drugs. (3) Results: A total of 170 SRs on SJS and TEN were identified, of which 32.9% were SJS and 67.1%—TEN. In total, 30% were pediatric SRs, 21.2%—SRs of the elderly. There were 12 lethal cases, and all cases were TEN. The leading culprit drugs were anti-infectives for systemic use and nervous system agents. The top 10 involved drugs are as follows: lamotrigine (23.5%), ibuprofen (12.9%), ceftriaxone (8.8%), amoxicillin and amoxicillin with beta-lactam inhibitors (8.8%), paracetamol (7.6%), carbamazepine (5.9%), azithromycin (4.1%), valproic acid (4.1%), omeprazole (3.5%), and levetiracetam (3.5%). (4) Conclusions: Our study was the first study in Russia aimed at the assessment of the structure of the drugs involved in SJS and TEN on the national level.

## 1. Introduction

The most severe forms of cutaneous adverse drug reactions (CADRs) are Stevens–Johnson syndrome (SJS) and toxic epidermal necrolysis (TEN) [1]. The time between the drug exposure and the first symptoms of severe CADRs may vary from 4 to 28 days [1], sometimes up to 8 weeks [2]. The severity and the extent of body surface area detachment (BSAD) are used to identify certain forms of severe CADR: SJS is diagnosed in <10% BSAD, SJS-TEN overlaps in 10–30% BSAD, and TEN in >30% BSAD [3,4].

The nature of reactions underlying SJS and TEN development is considered to be T-cell-mediated [5], with the principal role of T-cell receptors (TCRs). For example, public αβTCR demonstrated a binding affinity for carbamazepine, which is a well-known cause of SJS/TEN [6]. There are at least four hypotheses explaining the mechanisms of the drug-induced activation of T-cell response. For small-molecule drugs, the hapten concept is stated: small molecules can form antigenic hapten–carrier complexes with serum proteins; these complexes are recognized by certain HLA molecules and then presented to TCR. Pro-haptens are drugs, which may produce after biotransformation a protein-reactive derivative with the role of haptens [1,7,8]. Chemically inert drugs may directly bind T-cell receptors in non-covalent mode, or directly bind peptide-loaded major histocompatibility complex protein. This mechanism is known as a pharmacological interaction (p-i) concept [1,5]. The final concept is the altered peptide repertoire model. Based on this model, drugs bind directly to the HLA binding pocket altering the presentation of self-proteins to T-cells and leading to cytotoxic T-cell activation [1,5].

The characteristics of patients with SJS and TEN are well documented in the results of a pan-European multicenter cohort study (212 adult patients from 10 countries, the period from 1 January 2015 to 31 December 2019) [9]. The mean age of patients was 51.0 + 19.3 years, females were 63.7%, oral and ocular mucosal lesions (88.9% and 66.7%, correspondingly) were the most specific clinical manifestations, mean BSAD at presentation was 23.8%, and the 6-week mortality rate was 20.8% [9]. Among 23,265 patients hospitalized for SJS/TEN in the United States in the 2015 to 2020 period, 57.2% were female, and the median age was 58 years [10].

The incidence of SJS and TEN is low in the general population, though the lethality is high. The assessment of SJS and TEN epidemiology in the USA based on the total number of hospitalizations (392,302,031) for the period 2010–2020 revealed 51,040 (0.1%) hospitalizations for SJS/TEN, with a higher prevalence of SJS (73%, *n* = 37,283). A minority of the cases were for SJS-TEN overlap syndrome (15.3%, *n* = 7818) and TEN (14.0%, *n* = 7160) [10]. In the systematic review of PubMed/MEDLINE SSJ- and TEN-related case reports (analyzed period: 1980 to 2020; the number of analyzed cases—1059), the domination of TEN was demonstrated (56.8%, *n* = 602), SJS accounted for 36.0% (*n* = 381), and SJS-TEN overlap—7.2% (*n* = 76) [11]. The overall mortality rate was found to be 17.6%. In the case of SJS, it was 6.3%, in SJS-TEN overlap—21.1%, and in TEN—24.4% [11]. Since SJS and TEN pathogenesis involves immune system response, their incidence may be affected by the pandemic of COVID-19. Both the direct effects of SARS-CoV-2 on the immune system and the impact of immunosuppressing drugs like glucocorticoids and anticytokine monoclonal antibodies may be considered as important risk factors. In the study made in Australia, a seven-fold increase in SJS/TEN cases was observed since the COVID-19 pandemic; the authors reported nearly identical peaks for cumulative COVID-19 cases and cumulative SJS/TEN cases in 2022 [12].

Among the culprit pharmacological groups, antibacterial agents and anticonvulsants were the most frequently reported [13,14,15,16,17,18,19], followed by many others, including non-steroid anti-inflammatory drugs (NSAIDs), allopurinol, angiotensin-converting enzyme inhibitors, and antipsychotics [20,21,22,23,24]. Genetic predisposition is an important factor in SJS and TEN. The role of human leukocyte antigen (HLA) polymorphism in drug-induced SJS and TEN development was widely studied, especially for antiepileptic drugs. For lamotrigine, two alleles with protective properties against SJS and TEN were recently found, HLA-B*0702 and HLA-C*0702, and the presence of allele HLA-B*1502 was identified as a main risk factor for lamotrigine-induced SJS/TEN development [25]. A meta-analysis including 37 studies (the total number of participants was 51,422, of which cases were 7027 and controls—44,395) demonstrated the highest risk of carbamazepine-induced SJS/TEN in the presence of HLA-C (odds ratio, OR: 7.83; 95% credit interval, CI: 4.72 to 12.98), though significant risks were also detected for HLA-A, HLA-B, and HLA-DRB1. For lamotrigine-induced SJS/TEN, the highest risk was associated with HLA-A (OR: 2.38; 95% CI: 1.26 to 4.46) and HLA-B (OR: 2.79; 95% CI: 1.75 to 4.46); for phenytoin-induced SJS/TEN—HLA-A (OR: 3.47; 95% CI: 2.17 to 5.56), HLA-B (OR: 1.72; 95% CI: 1.38 to 2.15), and HLA-C (OR: 2.92; 95% CI: 1.77 to 4.83); for phenobarbital-induced SJS/TEN—HLA-A (OR: 6.98; 95% CI: 1.81 to 26.84), HLA-B (OR: 2.40; 95% CI: 1.39 to 4.17), and HLA-C (OR: 3.37; 95% CI: 1.03 to 11.01); and for zonisamide-induced SJS/TEN—HLA-A*02:07 (OR: 9.77; 95% CI: 3.07 to 31.1), HLA-B*46:01 (OR: 6.73; 95% CI: 2.12 to 21.36), and HLA-DRB1×08:03 (OR: 3.78; 95% CI: 1.20 to 11.97) [26]. Interesting data were obtained by Tangamornsuksan et al. (2020) who performed a systematic review and meta-analysis aimed at the determination of associations between HLA genotypes and the risks of cold medicine-induced SJS/TEN with severe ocular complications. The authors revealed the highest risk with HLA-A*0206, HLA-A*3303, HLA-B*4403, and HLA-C*0501 [27].

Racial and ethnic differences were widely demonstrated among patients with SJS and TEN which indicates a need to study these rare CADRs in different populations. Since the incidence of SJS and TEN is low and genetic predisposition is proven, data collection on the national level may give the most obvious information. Pharmacovigilance databases may be a valuable instrument to study SJS and TEN prevalence and the structure of suspected drugs. The objective of our study was to assess SJS and TEN characteristics in the general population of Russia, in the children, and in the elderly using data from the Russian National Pharmacovigilance database–Automatized Information System “Pharmacovigilance” (AIS).

## 2. Results

According to the hierarchical structure of MedDRA, SJS and TEN belong to two system organ classes (SOCs). The first SOC is “Skin and subcutaneous tissue disorders” with the corresponding high-level group term “Epidermal and dermal conditions”, and the second SOC is “Immune system disorders” with the corresponding high-level group term “Allergic conditions”. The number of SRs for the analyzed period (4 years and 9 months, from 1 April 2019 to 31 December 2023) for the first SOC was 15,813, and for the second—29,420. The total number of relevant spontaneous reports (SRs) on SJS and TEN registered in the AIS database was 170, which was 1.08% of the sample based on SOC “Skin and subcutaneous tissue disorders”, and 0.58% of the sample based on SOC “Immune system disorders”. The gained data indirectly indicate the rare development of SJS and TEN in the general population of Russia. SRs on SJS accounted for 32.9% (*n* = 56), and SRs on TEN—67.1% (*n* = 114).

All 170 SRs with SJS and TEN were estimated as serious adverse events (AEs). The distribution of SRs based on seriousness (as it was marked by reporters; in some SRs several criteria were chosen) is presented in Table 1.

The assessment of the demographic data indicated in the SRs revealed female gender in 61.8% (*n* = 105), male—34.1% (*n* = 58), and no data on gender—4.12% (*n* = 7) of the SRs. The distribution of patients by age group is presented in Table 2. The mean age was 36.8 ± 23.9 (min = 1 month, max = 93 years) years. Pediatric SRs on SJS and TEN (0–18 years) accounted for 30% (*n* = 51), and elderly (≥60 years)—21.2% (*n* = 36). Both SJS and TEN cases had peak values in the 19 to 59 year age group (Table 2). It should be noted that in the scale for assessing the severity and prognosis of SJS and TEN (SCORTEN) the age of patients > 40 is considered as an independent risk factor [28].

### 2.1. The Structure of Drugs Involved in SJS and TEN Development

One SR may include several suspected drugs, so the total number of drugs exceeded the number of SRs and amounted to 356. The mean number of suspected drugs per SR was 2.1 ± 1.97 (min = 1, max = 9).

There were 13 groups of drugs involved in SJS and TEN (identification was made using Anatomical Therapeutic Chemical (ATC) classification) (Table 3). They covered 99.5% (*n* = 354) of drugs. The additional 0.5% (*n* = 2) were biologically active dietary supplements not coded in ATC.

**Figure 1:**
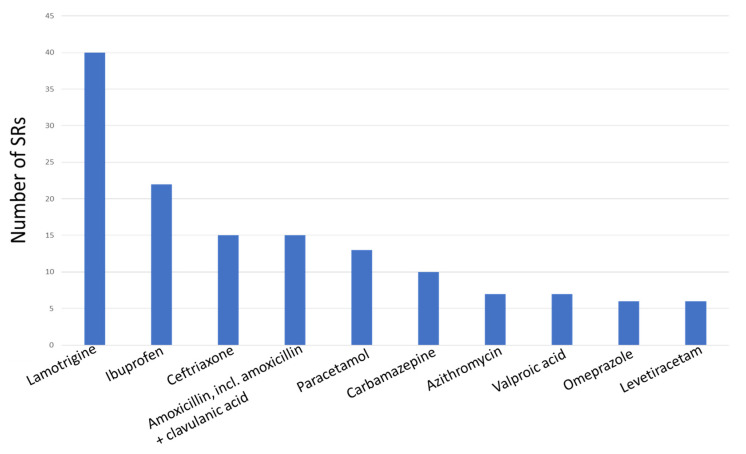
Top 10 drugs involved in SJS and TEN.

The next step of the analysis included the identification of the top 10 drugs involved in SJS and TEN. The absolute leader was lamotrigine, indicated in 23.5% of the SRs (*n* = 40), followed by ibuprofen—12.9% (*n* = 22), ceftriaxone—8.8% (*n* = 15), and amoxicillin, including amoxicillin in combination with clavulanic acid—8.8% (*n* = 15). The total structure of the top 10 drugs is presented in Figure 1.

#### 2.1.1. ATC Level 1 Group J Drugs Involved in SJS and TEN

The total number of drugs in the group J was 103 (28.9% of the total number of suspected drugs). Among them, 75 drugs (72.8% of group J drugs; 20.5% of all suspected drugs) were from the subgroup J01—antibacterials for systemic use. The absolute leaders were beta-lactams (61.3%, *n* = 46), and the main contribution was made by the third-generation cephalosporins (32%, *n* = 24). The most frequently reported drug from the J01 group was ceftriaxone (20%, *n* = 15). The total structure of the J01 drugs involved in SJS and TEN development is displayed in Table 4.

The analysis of the remaining subgroups within the J group revealed a considerable share of J05, antivirals for systemic use, and they accounted for up to 20.4% (*n* = 21) of the J group. The leader within the J05 subgroup was acyclovir (19%, *n* = 4). The J05 drugs involved in SJS and TEN are presented in Table 5.

#### 2.1.2. ATC Level 1 Group N Drugs Involved in SJS and TEN

The total number of the N group drugs was 102 (28.7% of the total number of suspected drugs). Most (61.8%, *n* = 63) belonged to the subgroup N03—antiepileptics (the leaders were lamotrigine (39.2%, *n* = 40), carbamazepine (9.8%, *n* = 10), and valproic acid (6.9%, *n* = 7)). High rates were also reported for the N02 subgroup, analgesics (18.6%, *n* = 19). Data on the N group drugs are given in Table 6.

### 2.2. SJS and TEN in Pediatric SRs

The total number of pediatric SRs on SJS and TEN was 51 (30.0% of the total SRs). The mean age of patients was 8.9 ± 5.3 years (min—1 month, max—17 years), female gender was indicated in 56.9% (*n* = 29), male—37.3% (*n* = 19), and no data on gender—5.9% (*n* = 3).

SJS was reported in 18 cases (35.3%), mean age was 9.0 ± 5.8 (min—1 month; max—17 years), females were 50% (*n* = 9), males—33.3% (*n* = 6), and unknown gender—16.7% (*n* = 3).

The diagnosis of TEN was indicated in 33 (64.7%) SRs. The mean age of patients was 10 ± 5 years (min—3 months; max—17 years), females were 60.6% (*n* = 20), and males—39.4% (*n* = 13).

The total number of suspected drugs in pediatric SRs was 119, and the mean number of drugs per SR was 2.3 ± 1.6 (min = 1, max = 6). In the structure of the drugs involved, the most common ATC groups were N (36.13%, *n* = 43), J (25.21%, *n* = 30), and M (18.49%, *n* = 22).

Comparing the drugs involved in SJS and TEN in children with those in the general population we revealed a higher proportion of antiepileptics reported in pediatric SRs (26.9%, 32/119 vs. 17.7%, 63/356). Antibacterials were less frequently reported in children compared with the general population (21.0%, 25/119 vs. 28.9%, 103/356). The drugs involved in SJS and TEN in children are indicated in Table 7.

### 2.3. SJS and TEN in the Elderly SRs

Patients over 60 years old accounted for 21.2% of all SJS and TEN SRs (*n* = 36); SJS was indicated in 10 SRs (27.8%), and TEN—26 (72.2%). The mean age of patients was 69.7 ± 8.0 (min—60, max—93) years, females were 55.6% (*n* = 20), and males were 44.4 (*n* = 16).

The total number of suspected drugs in the SRs of the elderly was 48, with a mean number of drugs per SR of 1.4 ± 0.7 (min = 1, max = 3), which was less than in the general population (2.1). The following ATC level 1 groups were found to be leaders in the elderly: J (39.6%, *n* = 19) and L (20.8, *n* = 10). The proportion of the J group drugs exceeded that of the general population (39.6%, 19/48 vs. 29.1%, 103/354). A specific feature of the elderly was the high rate of antineoplastic drugs involved in SJS and TEN (20.8%, 10/48 vs. 5.6%, 20/356 in the general population). This fact may be explained by a higher prevalence of cancer among older patients. The proportion of reported antiepileptics in the elderly was significantly less than in the general population (8.33%, 4/48 vs. 17.7%, 63/356). The drugs involved in SJS and TEN in the elderly are demonstrated in Table 8.

**Figure 2:**
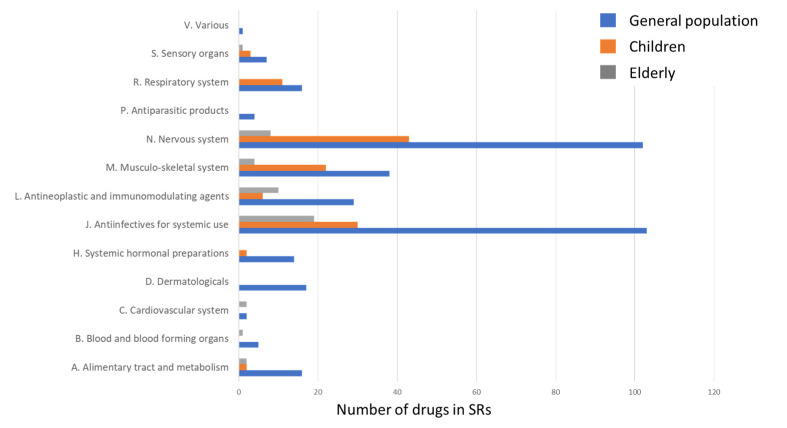
ATC level 1 groups involved in SJS and TEN in the general population, children, and elderly patients.

The analysis of ATC level 1 groups involved in SJS and TEN in the elderly, children, and in the general population made it clear that age is associated with a different structure of leading causative agents (Figure 2). The following ATC groups were not reported in the SRs of the elderly: D, H, P, R, and V.

### 2.4. Lethal Cases

Lethal outcomes were reported in 7.1% (*n* = 12) of the SRs, and all cases were TEN. The mean age of patients was 52.0 ± 22.3 (min = 15, max = 84) years, and females were 50% (*n* = 6). Among the SRs with lethal outcomes 19 suspected drugs were reported, and the mean number of drugs per SR was 1.6 ± 0.9 (min = 1, max = 3).

There was only one death in children and five lethal cases in the elderly. A fatal TEN episode was reported in a 15-year-old boy, and the suspected drug was lamotrigine prescribed to treat anxiety–depressive syndrome. There were minor developmental anomalies (abnormal cardiac chord and mitral valve prolapse) and cardiac arrhythmias in the medical history of this patient. The first symptoms of TEN appeared in 2 weeks after lamotrigine treatment started. Despite the discontinuation of the causative drug and intensive therapy, sepsis with multiple organ failure developed, and cardiac arrest led to death.

The analysis of the SRs of the elderly with fatal outcomes revealed a mean age of 72.0 ± 9.1 (min—62, max—84) years, females were 60% (*n* = 3), and males—40% (*n* = 2). Ibrutinib, prescribed for the treatment of blood cancer, was considered as suspected drug in two SRs, antibiotics from the cephalosporin group in another two SRs (cefazolin, *n* = 1; ceftriaxone, *n* = 1), and a drug for the treatment of arterial hypertension (perindopril) in one SR.

**Figure 3:**
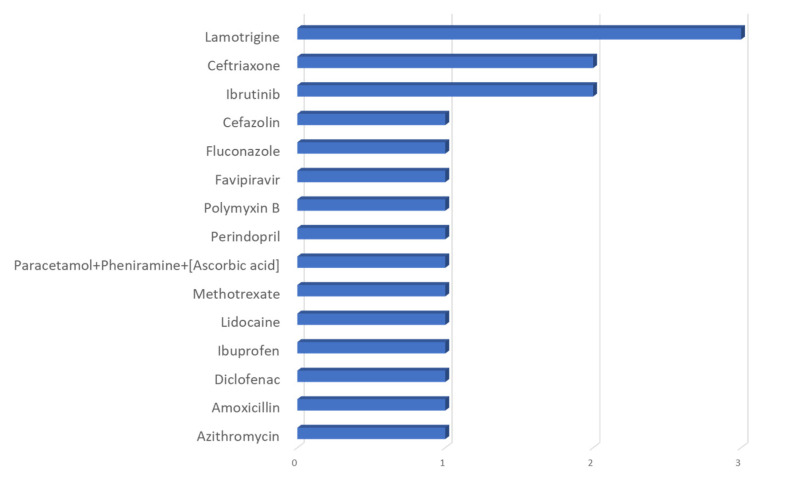
Contribution of different drugs in fatal TEN.

The highest frequency of reporting among all the fatal SRs was observed for lamotrigine (*n* = 3), ceftriaxone (*n* = 2), and ibrutinib (*n* = 2). Figure 3 demonstrates the contribution of different drugs in fatal TEN.

## 3. Discussion

Our results revealed an approximately two-fold predominance of TEN SRs compared with SJS SRs (67.1% vs. 32.9%). It may be the result of the underreporting of mild SJS cases, including situations when SJS was not well recognized by a doctor. The results of many published studies indicate a higher prevalence of SJS (92.6% [29], 89.4% [30], 80.4% [31], and 73% [32]), though there are other works demonstrating a greater proportion of TEN cases among severe CADRs. The analysis of a network of full-text databases in China accompanied by hospital admission database analysis (from 2006 to 2016) defined 166 cases of SJS and TEN, of which 56.6% were TEN, 42.2%—SJS, and 1.2%—SJS-TEN overlap [33]. SJS and TEN cases published in the MEDLINE database between 1980 and 2020 were analyzed by Wang L et al. (2022) and the results revealed TEN in 56.8% (*n* = 602), SJS-TEN overlap in 7.2% (*n* = 76), and SJS in 36% (*n* = 381) [11]. Estimating BSAD in the medical records of patients with SJS and TEN collected for the period 1999 to 2014 in the public healthcare system of the Federal District, Brazil, Arantes et al. (2017) demonstrated BSAD > 30% in 76.2% (16 cases out of 21 reported in the referral hospital) [34]. The results of a retrospective study of the medical records of patients with SJS and TEN performed in a clinic in Turkey (period from January 2008 to June 2019) revealed TEN in 57.6%, SJS in 33.3%, and SJS-TEN overlap in 9.1% [35]. The analysis of SJS and TEN patients’ data (USA, Loyola University Medical Center Burn Unit, period from 2010 to 2019) indicated TEN to be the most prevalent (48.1%), followed by SJS (33.6%) and SJS-TEN overlap (18.3%) [18]. A retrospective study including the data from all patients hospitalized with SJS and TEN in a hospital in Saudi Arabia from 2014 to 2019 (*n* = 10) reported six cases were TEN [36]. A prospective observational study conducted in a tertiary care hospital in South India (from January 2016 to June 2017) revealed TEN in 64.7%, and SJS and SJS-TEN in 17.6% each [37].

Our results demonstrated that 30% of the SJS/TEN were in children and 21.2%—the elderly. Older age was identified as a risk factor for SJS/TEN development in the population-based longitudinal cohort study which included 2,398,393 Japanese individuals. The highest hazard ratio, HR, was demonstrated for 70 to <80 years old patients; it was 2.91 (95% CI 1.57–5.23; *p* < 0.001), and other risk factors were the presence of systemic autoimmune disease (HR 1.8; 95% CI 1.07–3.03; *p* = 0.027), peripheral vascular disease (HR 1.76; 95% CL 1.24–2.51; *p* = 0.002), and type 2 diabetes (HR 1.53; 95% CI 1.01–2.32; *p* = 0.043) [38].

The mortality rate in TEN typically exceeds that of SJS. All 12 fatal cases reported in our study were TEN (10.5% of all TEN cases and 7.1% of all SJS and TEN cases), and the mean age of patients was 52.0 ± 22.3 (min = 15, max = 84) years. A retrospective study of patients with SJS and TEN performed in a clinical center in India (analyzed period 2010–2020) revealed no lethal cases for SJS and 16% for TEN [39]. According to the analysis of the database on hospital inpatient stays in the United States (from 2010 to 2020) mortality rate for TEN was 15.3%, for SJS—5.4%, and for SJS-TEN overlap—14.4% [10]. A nationwide survey performed in Japan (2016–2018) reported a 29.9% mortality rate for TEN and only 4.1% for SJS [40]. Nearly the same value for TEN mortality was demonstrated in the study including data from hospitals in the Italian Lombardy region—29.4%, while for SJS it was estimated at the level of 16.9% [41]. A retrospective analysis of 59 TEN cases reported in Yokohama City University Hospital and Yokohama City University Medical Center between January 2000 and March 2020 revealed a mortality rate of 13.6% (*n* = 8) [42]. Higher rates of SJS/TEN mortality were demonstrated in the USA: for the period from 2004 to 2021 there were 24,976 cases of SJS/TEN in the Food and Drug Administration Adverse Event Reporting System (FAERS) database, and 19.53% were fatal [43].

According to our results, 41.7% (*n* = 5) of the fatal SRs were from the elderly, and the total share of the fatal SRs of patients >45 years was 66.7% (*n* = 8). The published data indicate older age to be one of the potential risk factors for increased mortality in the SJS and TEN population [10]. Noe M.H. et al. (2019) developed a model of risk prediction for in-hospital mortality among patients with SJS/TEN and considered age as its independent predictor [44]. We have demonstrated lethal outcomes in 13.9% of the SRs of the elderly (*n* = 5) and only in 2% of the SRs of children (*n* = 1). The analysis of the United States 2016 Kids’ Inpatient database revealed 153 pediatric SJS/TEN cases with a mortality rate of 4.81% [45]. These data prove the less severity of CADRs in children.

Our results indicated that anti-infectives for systemic use were the most frequently reported drugs for SJS and TEN in the general population, with a predominance of antibacterials for systemic use. Beta-lactams (61.3%, *n* = 46) made the greatest contribution and the subgroup of third-generation cephalosporins (32%, *n* = 24) was the most frequently reported. The fact of the wide involvement of antibiotics in SJS and TEN development was proved by the results of a systematic review of PubMed/MEDLINE case reports from 1980 to 2020 (*n* = 1059). The authors demonstrated that 26.9% of the cases were related to antibiotics, while other groups were less frequently reported (anticonvulsants—18.5%, analgesics/anesthetics—11.9%, and antineoplastics—11.3%) [11]. In a population of hospitalized patients with TEN (Czech Republic and Slovakia, period from 2000 to 2015) antibacterials were revealed as a cause in 46.2% (most drugs were aminopenicillins) [46]. In the study including data from 7,337,778 individuals in Hong Kong (2016 to 2021), anti-infective agents were determined as the main causative group resulting in SJS induction (43.1%), and beta-lactams were the most common drugs (20.4%) [47]. A systematic review and meta-analysis of 38 studies with 2917 patients (MEDLINE and Embase databases) determined that the pooled proportion of antibiotics associated with SJS and TEN was 28% (95% credit interval, CI, 24–33%). The highest association was proved for sulfonamides (32% of cases; 95% CI, 22–44%), penicillins (22%; 95% CI, 17–28%), and cephalosporins (11%; 95% CI, 6–17%) [17].

The assessment of the risk of SJS and TEN associated with different antibiotic classes performed in Japan demonstrated maximum odds ratios for lincomycins (33.00 [95% CI, 3.74–4332.05]), sulfamethoxazole trimethoprim (21.20 [6.73–105.98]), penicillins (14.39 [6.95–34.21]), glycopeptides (14.37 [3.17–136.10]), cephalosporins (7.06 [4.25–12.21]), aminoglycosides (6.55 [1.97–26.84]), quinolones (5.98 [3.34–11.20]), fosfomycin (5.40 [1.20–30.97]), carbapenems (5.09 [1.85–15.64]), tetracyclines (4.95 [1.78–15.27]), and macrolides (3.78 [2.13–6.83]) [48]. Sulfonamides (mainly sulfamethoxazole trimethoprim) were reported by many researchers among the most common culprit drugs for SJS and TEN among anti-infectives. Our data revealed only one case of SJS associated with sulfanilamide and one case associated with sulfamethoxazole trimethoprim; a possible explanation of such a low prevalence may be related to the fact of a limited prescription of sulfonamides in Russia due to a high rate of antibiotic resistance [49,50].

The second leading group of drugs involved in SJS and TEN based on our results was the N group (28.8% of all the drugs involved), with antiepileptics accounting for up to 63.7% and lamotrigine being the absolute leader. Antiepileptics are well-known causes of SJS and TEN. They accounted for up to 19.37% of all SJS and TEN reports retrieved from the FAERS database from 2004 to 2021 [43]. There are a lot of studies identifying the high risk of SJS and TEN associated with antiepileptics. The analysis of FAERS from July 2014 through December 2017 revealed six antiepileptics whose use increases SJS and TEN risks greater than 20 times compared with non-users. These drugs were zonisamide (reporting odds ratios (ROR): 70.2, 95% CI 33.1–148.7; proportional reporting ratios (PRR): 68.7, 95% CI 32.9–143.5), rufinamide (ROR 60.0, 95% CI 8.3–433.5; PRR 58.9, 95% CI 8.4–411.5), clorazepate (ROR 56.0, 95% CI 7.8–404.1; PRR 55.1, 95% CI 7.8–385.0), lamotrigine (ROR 53.0, 95% CI 43.2–64.9; PRR 52.2, 95% CI 42.7–63.7), phenytoin (ROR 26.3, 95% CI 15.5–44.7; PRR 26.1, 95% CI 15.4–44.2), and carbamazepine (ROR 24.5, 95% CI 16.0–37.5; PRR 24.3, 95% CI 16.0–37.1) [51]. Analyzing the medical records of patients with SJS and TEN admitted to Loyola University Medical Center Burn Unit (USA) in the period 2000 to 2019, the authors concluded that antiepileptics were the main causative drugs (30% of the cases) followed by trimethoprim-sulfamethoxazole (19%) [18]. The results gained in South Korea revealed such antiepileptics as carbamazepine and lamotrigine among the most frequently suspected culprit drugs [52]. Fukasawa et al. (2021) performed an estimation of ORs for SJS/TEN for each anticonvulsant use versus non-use in the Japanese population and demonstrated the highest risks for new carbamazepine users (OR 68.00) and lamotrigine users (OR 36.00) [53]. Another study analyzing the database of Clalit Health Services (Israel, period from 1 January 2008 to 30 June 2019) demonstrated similar high risks of SJS and TEN for the new users of antiepileptics. For phenytoin, there were 3.56 SJS/TEN cases per 10,000 new users, and for lamotrigine—2.82, which dramatically exceeded the value for another common culprit drug—allopurinol (1.10) [16]. Chiang et al. (2024) demonstrated the N group drugs as the second most frequent culprits of SJS (22.9%) after anti-infectives [47]. Among patients with SJS and TEN hospitalized in Pusan National University Hospital (South Korea) between 2008 and 2019, antiepileptics were in the third place (18.5%) after antibiotics (30.4%) and allopurinol (21.7%) [13].

The comparison of the structure of suspected drugs in the pediatric and general populations revealed the dramatic predominance of N group drugs (36.1% vs. 28.8% in general population), with antiepileptics accounting for up to 26.9% of the total drugs involved in SJS and TEN in children. Our results are in line with the published data: antiepileptics were the main culprit drugs, reported in 45.2% of the cases of SJS and TEN in the pediatric population analyzed in Iran (retrospective study of patients’ data from two hospitals for 5 year period) [54]. The identification of pediatric patients with SJS and TEN at the Shriner’s Burn Hospital in Galveston, Texas, USA (period from 1990 to 2015) revealed phenytoin and lamotrigine used concomitantly with valproic acid among the most common culprit drugs, and antiepileptics approved after 1990 (lamotrigine, clobazam, and zonisamide) were the cause in 25.5% of the cases [55]. A 20-year database review of all children diagnosed with SJS and TEN at the King Chulalongkorn Memorial Hospital, Thailand, revealed that antiepileptics were the leading culprit group (36.1%), followed by antibiotics (25.0%). For the subgroup of SJS only, antibiotics were the leading group (35.0%), and for the SJS-TEN overlap half of all cases were caused by antiepileptics (50.0%), while antibiotics were responsible only for 12.5% [56]. Another study used data from a single clinical center in North America (from 2008 to 2018) and defined antiepileptics as the second cause of SJS and TEN in children (31.3%) after antibiotics (56.3%) [57].

The L group drugs (antineoplastic and immunomodulating agents) were found to be the second most frequently reported ATC group after anti-infectives for systemic use in the elderly in our study. This fact may be explained by a higher incidence of oncological diseases among the elderly compared with adults and children, and thereby a greater consumption of antineoplastic agents. Published data demonstrated wide involvement of targeted anticancer drugs and immunotherapies in severe CADR development, including SJS and TEN cases [58]. FAERS database analysis revealed antineoplastic agents among the top drug classes associated with SJS/TEN deaths [43]. For the Japanese population, anticancer drugs were the more frequent causes of SJS and TEN in patients older than 80 years compared with patients younger than 50 years [59].

In our study group, M drugs accounted for up to 10.7% and NSAIDs were the most common among them. Ibuprofen was the second top drug identified with 12.9% of the SRs reporting it as a suspected drug. NSAIDs were demonstrated to be associated with a high risk of SJS and TEN. The analysis of the database derived from Clinical Practice Research Datalink (United Kingdom) allowed the authors to identify an association between certain drug use and SJS and TEN risk. OR for cyclooxygenase-2 inhibitors was 24.19 (95% CI, 2.91–200.92), and the absolute risks of SJS and TEN was 1.9–4.3 per 100,000 new users for cyclooxygenase-2 inhibitors [60]. The leading role of ibuprofen in SJS and TEN development was proved by the analysis of the data retrieved from the FAERS database from January 2004 to March 2021. Ibuprofen revealed the highest association with SJS based on the highest ROR (ROR = 7.06, 95% two-sided CI 6.59–7.56) and was the most frequently reported NSAID in SJS and TEN [21]. Fei et al. (2023) listed valdecoxib and celecoxib among the top 50 drugs associated with more than 60% of the SJS/TEN reactions in the United States, while diclofenac and acetylsalicylic acid were among the drugs associated with a minority of the SJS and TEN cases (less than 10% of the SJS/TEN reactions in the United States) [43].

The limitations of our study are typical limitations specific to the spontaneous reporting method and include the following: underreporting, variable reporting rate considering different settings and time periods, the low quality of some spontaneous reports, and inability to establish the frequency of ADRs, since the total size of the population of drug users in unknown.

On the other hand, an important advantage of the spontaneous reporting method is the ability to obtain information on the use of drugs in all groups of patients (age, social groups, groups with and without comorbidities, etc.) in real clinical practice over a long time period, which is especially important when studying rare ADRs.

Differences in the structure of the drugs involved in the development of SJS and TEN in different regions of the world and ethnic groups may correlate with the established role of genetic factors in the occurrence of CADRs, as well as with the peculiarities of the prescription patterns of drugs in different countries and ethnic groups [61,62,63,64]. The geography of HLA-associated serious CADRS was shown to be dependent on the world distribution of the carriage rate of certain HLA alleles [65,66]. International Collaboration on SJS/TEN With Severe Ocular Complications indicated cold medicines, including NSAIDs, as the main group of drugs inducing studied pathology among Japanese patients. Both for Japanese and Korean patients HLA-A*02:06 was implicated, and in Japanese-, Indian-, and European ancestry Brazilian patients—HLA-B*44:03 [67]. Non-HLA factors also may contribute to SJS and TEN. Recently, pharmacogenetic variants in aldoketoreductase 1C (AKR1C)2–4 demonstrated their possible role in the SJS-TEN overlap [68].

The given data suggest an urgent need to study SJS and TEN drug triggers at the national level together with the perspectives of further genetic studies. Our study is the first step of wider research which will be dedicated to the assessment of the national features of SJS and TEN in the Russian population. The next planned step is genetic testing of patients hospitalized with SJS and TEN and the identification of HLA alleles associated with SJS/TEN risks in the Russian population. Considering the obtained data on the structure of the involved drugs, we believe that the results will help us to develop a knowledge system aimed at assisting healthcare professionals to minimize SJS and TEN risks. For example, the antibiotic most involved in SJS and TEN in our study was ceftriaxone, and we can recommend the limitation of its wide use in outpatients (it is common in Russian clinical practice both for adults and children), which is accompanied by a late recognition of pathological state and the absence of rescue medicines and physician’s monitoring, leading to a higher risk of lethal outcome.

## 4. Materials and Methods

Federal Service for Surveillance in Healthcare (Roszdravnadzor) is responsible for AE data collection in the Russian Federation. All SRs were entered into the AIS “Pharmacovigilance” database, whose structure, functioning, and management comply with the ICH E2B (R3) standard [69]. MedDRA version 25.0 was used in AIS [70]. Causality assessment in the AIS database was made by the authors using the built-in WHO algorithm and Naranjo algorithm [71]. According to the Naranjo algorithm, only ADRs with a high (certain, probable, and possible) causal relationship were included in the study. In Russia, adverse events reporting may be performed by healthcare professionals (in fact, the absolute majority of cases), the workers of pharmaceutical companies, patients, or their representatives.

The study design was a retrospective descriptive pharmacoepidemiologic study. The object of the study—SRs registered in the AIS in the period from 1 April 2019 to 31 December 2023. Inclusion criteria: “Stevens-Johnson Syndrome” or “Toxic Epidermal Necrolysis” indicated in SRs; SRs collected on the territory of the Russian Federation. Duplicates and invalid SRs were excluded. The validity of the SRs was determined in accordance with paragraph 407 of the “Rules of Good Pharmacovigilance Practice of the EAEU” [72], stating the necessity of all the following four elements in SRs: identifiable reporter; identifiable patient; at least one suspected drug; and at least one suspected ADR. In the absence of any of these four elements, the SR was marked as invalid. The authors assessed the presence of complete information describing the suspected drug, developed ADR, patient (gender, age, weight, diagnosis, etc., with the exception of the patient’s personal data), and reporter (the last criterion is automatically confirmed when the report is entered into the AIS database). The severity of the ADR was assessed in accordance with paragraph 2 of the “Rules of Good Pharmacovigilance Practice of the EAEU” [72].

**Figure 4:**
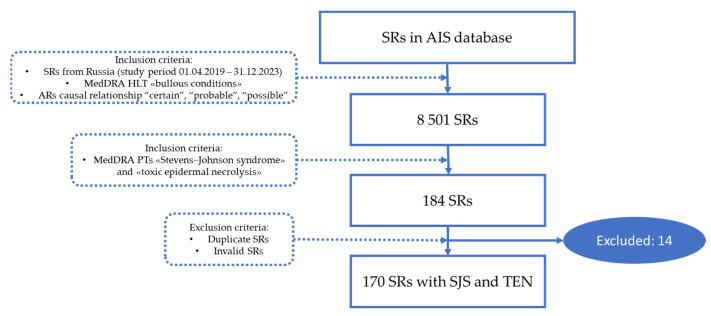
Flowchart of SR selection from AIS “Pharmacovigilance” (AR—adverse reaction, HLT—high level term; PT—preferred term; SR—spontaneous report).

The flowchart of SR selection from the AIS database is presented in Figure 4.

Suspected drugs were identified by INN, and the division into groups was carried out in accordance with the international Anatomical Therapeutic Chemical Classification.

Statistical analysis: Patient demographics were assessed based on SR data. Statistical data processing was carried out using the Microsoft Excel 2019 software. Descriptive statistics were used for all analyzed indicators; qualitative variables were described by absolute (n) and relative (%) values. Statistical analysis methods were not used to determine the reliability of the differences in the results obtained, since the method of SR does not allow estimating the size of the population.

The following definitions were used in our study [73]:

*“Adverse reaction—A response to a medicinal product, which is noxious and unintended. Adverse reaction may arise from use of the product within or outside the terms of the marketing authorization or from occupational exposure. Use outside the marketing authorization includes off-label use, overdose, misuse, abuse, and medication errors.”*

*“Causality—In accordance with ICH-E2A, the definition of an adverse reaction implies at least a reasonable possibility of a causal relationship between a suspected medicinal product and an adverse event. An adverse reaction, in contrast to an adverse event, is characterized by the fact that a causal relationship between a medicinal product and an occurrence is suspected. For regulatory reporting purposes, as detailed in ICH-E2D, if an event is spontaneously reported, even if the relationship is unknown or unstated, it meets the definition of an adverse reaction. Therefore, all spontaneous reports notified by healthcare professionals or consumers are considered suspected adverse reactions, since they convey the suspicions of the primary sources, unless the reporters specifically state that they believe the events to be unrelated or that a causal relationship can be excluded.”*

*“A spontaneous report is an unsolicited communication by a healthcare professional, or consumer to a competent authority, marketing authorisation holder or other organization (e.g., regional pharmacovigilance center, poison control center) that describes one or more suspected adverse reactions in a patient who was given one or more medicinal products. It does not derive from a study or any organized data collection systems.”*

## 5. Conclusions

National pharmacovigilance database analysis allows us to identify the predominance of TEN cases among the registered SRs in Russia. In the general population and in the elderly, most of the SJS and TEN reports were related to the group J drugs, and in children the leading group was N. The top 10 drugs involved in SJS and TEN in our study were lamotrigine (23.5%), ibuprofen (12.9%), ceftriaxone (8.8%), amoxicillin and amoxicillin in combination with beta-lactam inhibitors (8.8%), paracetamol (7.6%), carbamazepine (5.9%), azithromycin (4.1%), valproic acid (4.1%), omeprazole (3.5%), and levetiracetam (3.5%). The estimation of demographic data revealed a high proportion of pediatric SRs on SJS and TEN, 30%, and less SRs were from the elderly (21.2%). Our study demonstrated lethal cases only in TEN (7.06% of all SJS and TEN SRs or 10.5% of all TEN SRs), and the patients who died were older than the general population with SJS and TEN.

## Figures and Tables

**Table 1:** SR distribution based on seriousness criteria.

Criterion	N (Total—170)	%
AEs resulted in death	12	7.1
Life-threatening AEs + AEs requiring or prolonging hospitalization (both criteria chosen)	36	21.2
Life-threatening AEs	28	16.5
AEs causing persistent or significant disability or incapacity + AEs requiring or prolonging hospitalization (both criteria chosen)	1	0.6
AEs requiring or prolonging hospitalization	69	40.5
Other conditions judged to represent significant hazards	24	14.1

**Table 2:** Age distribution of patients with SJS and TEN.

Age Group	N (Total—170)	%	SJS N (Total—56) (%)	TEN N (Total—114) (%)
0–1 (infants)	4	2.4	1 (1.8)	3 (2.6)
>1–3 (toddlers)	8	4.7	5 (8.9)	3 (2.6)
4–12 (childhood)	23	13.5	7 (12.5)	16 (14.0)
13–18 years (adolescence)	16	9.4	5 (8.9)	11 (9.6)
19–59 years (adults)	77	45.3	23 (41.1)	54 (47.4)
60–74 years (youngest-old)	29	17.1	8 (14.3)	21 (18.4)
75–89 years (middle-old)	5	2.9	1 (1.8)	4 (3.5)
≥85 years (oldest-old)	2	1.2	1 (1.8)	1 (0.9)
No data	6	3.5	5 (8.9)	1 (0.9)

**Table 3:** Drugs involved in SJS and TEN.

ATC 1 Level Group	N (Total—354)	%
A. Alimentary tract and metabolism	16	4.5
B. Blood and blood-forming organs	5	1.4
C. Cardiovascular system	2	0.6
D. Dermatologicals	17	4.8
H. Systemic hormonal preparations	14	3.9
J. Anti-infectives for systemic use	103	29.1
L. Antineoplastic and immunomodulating agents	29	8.2
M. Musculo-skeletal system	38	10.7
N. Nervous system	102	28.8
P. Antiparasitic products	4	1.1
R. Respiratory system	16	4.5
S. Sensory organs	7	2.0
V. Various	1	0.3

**Table 4:** J01 drugs involved in SJS and TEN.

Drugs	N (Total—75)	%
J01A Tetracyclines (Tigecycline)	2	2.7
J01B Amphenicols (Chloramphenicol)	3	4.0
J01C Beta-lactams, penicillins	16	21.3
Amoxicillin	8	10.7
Ampicillin + sulbactam	1	1.3
Amoxicillin + sulbactam	1	2.7
Amoxicillin + clavulanic acid	6	8
J01D Other beta-lactam antibiotics	30	40.0
J01DB First-generation cephalosporins (Cefazolin)	1	1.3
J01DC Second-generation cephalosporins (Cefuroxime)	1	1.3
J01DD Third-generation cephalosporins	24	32.0
Cefotaxime	2	2.7
Ceftriaxone	15	20.0
Cefixime	4	5.3
Cefoperazone + sulbactam	3	4.0
J01DH Carbapenems	4	5.3
Meropenem	3	4.0
Imipenem + cilastatin	1	1.3
J01E Sulfonamides and trimethoprim	2	2.7
J01EB Short-acting sulfonamides (Sulfanilamide)	1	1.3
J01EE Combinations of sulfonamides and trimethoprim, including derivatives (Trimethoprim/sulfamethoxazole)	1	1.3
J01FA Macrolides	11	14.7
Erythromycin	2	2.7
Clarithromycin	2	2.7
Azithromycin	7	9.3
J01GB Other aminoglycosides (Kanamycin)	1	1.3
J01MA Fluoroquinolones	6	8.0
Ciprofloxacin	2	2.7
Levofloxacin	3	4.0
Moxifloxacin	1	1.3
J01X Other antibacterials	4	5.3
J01XA Glycopeptide antibacterials (Vancomycin)	3	4.0
J01XB Polymyxins (Polymyxin B)	1	1.3

**Table 5:** J05 drugs involved in SJS and TEN.

Drugs	N (Total—21)	%
J05AB Nucleosides and nucleotides excluding reverse transcriptase inhibitors	5	23.8
Acyclovir	4	19
Valgancyclovir	1	4.8
J05AC Cyclic amines (Rimantadine)	1	4.8
J05AE Protease inhibitors	2	9.5
Ritonavir	1	4.8
Darunavir	1	4.8
J05AF Nucleoside and nucleotide reverse transcriptase inhibitors	6	28.6
Lamivudine	3	14.3
Tenofovir disoproxil	3	14.3
J05AG Non-nucleoside reverse transcriptase inhibitors	2	9.5
Nevirapine	1	4.8
Efavirenz	1	4.8
J05AX Other antivirals	5	23.8
Riamilovir	1	4.8
Umifenovir	1	4.8
Favipiravir	3	14.3

**Table 6:** Group N drugs involved in SJS and TEN.

Drugs	N (Total—102)	%
N01B Anesthetics, local	2	2.0
N01BA Esters of aminobenzoic acid (Procaine)	1	1.0
N01BB Amides (Lidocaine)	1	1.0
N02B Other analgesics and antipyretics	19	18.6
N02BA Salicylic acid and derivatives (Acetylsalicylic acid)	2	2.0
N02BE Anilides	17	16.7
N02BE01 Paracetamol	13	12.7
N02BE51 Paracetamol, combinations excluding psycholeptics	4	3.9
N03 Antiepileptics	65	63.7
N03AA Barbiturates and derivatives (Phenobarbital)	2	2.0
N03AF Carboxamide derivatives (Carbamazepine)	10	9.8
N03AG Fatty acid derivatives (Valproic acid)	7	6.9
N03AX Other antiepileptics	46	45.1
Lamotrigine	40	39.2
Levetiracetam	6	5.9
N04AA Tertiary amines (Trihexyphenidyl)	1	1.0
N04BB Adamantane derivatives (Amantadine)	1	1.0
N05 Psycholeptics	8	7.8
N05A Antipsychotics	6	5.9
N05AD Butyrophenone derivatives	2	2.0
Haloperidol	1	1.0
Droperidol	1	1.0
N05AH Diazepines, oxazepines, thiazepines, and oxepines (Quetiapine)	2	2.0
N05AL Benzamides (Tiapride)	1	1.0
N05AX Other antipsychotics (Paliperidone)	1	1.0
N05B Anxiolytics	2	2.0
N05BA Benzodiazepine derivatives (Alprazolam)	1	1.0
N05BX Other anxiolytics (Phenazepam)	1	1.0
N06 Psychoanaleptics	4	3.9
N06A Antidepressants	3	2.9
N06AA Non-selective monoamine reuptake inhibitors (Amitriptyline)	1	1.0
N06AB Selective serotonin reuptake inhibitors (Sertraline)	2	2.0
N06BX Other psychostimulants and nootropics (bovine cerebral cortex polypeptides)	1	1.0
N07XX Other nervous system drugs (Ethylmethylhydroxypyridine succinate)	2	2.0

ADHD—Attention Deficit Hyperactivity Disorder.

**Table 7:** Drugs involved in SJS and TEN in children.

Drugs	N (Total—119)	%
A. Alimentary tract and metabolism	2	1.7
A02 Drugs for acid-related disorders (Omeprazole)	1	0.8
A12 Mineral supplements (magnesium (different salts in combination))	1	0.8
H Systemic hormonal preparations, excluding sex hormones and insulins	2	1.7
H02 Corticosteroids for systemic use (Prednisolone)	1	0.8
H03 Thyroid therapy (Potassium iodide)	1	0.8
J. Anti-infectives for systemic use	30	25.2
J01 Antibacterials for systemic use	25	21
J01B Amphenicols (Chloramphenicol)	3	2.5
J01C Beta-lactam antibiotics, penicillins	9	7.6
Amoxicillin	4	3.4
Amoxicillin + clavulanic acid	5	4.2
J01D Other beta-lactam antibiotics	10	8.4
J01DD Third-generation cephalosporins	10	8.4
Cefotaxime	1	0.8
Ceftriaxone	5	4.2
Cefixime	3	2.5
Cefoperazone + sulbactam	1	0.8
J01FA Macrolides (Azithromycin)	2	1.7
J01MA Fluoroquinolones (Levofloxacin)	1	0.8
J05 Antivirals for systemic use	3	2.5
J05AB Nucleosides and nucleotides excluding reverse transcriptase inhibitors (Acyclovir)	2	1.7
J05AX Other antivirals (Umifenovir)	1	0.8
J07 Vaccines	2	1.7
L. Antineoplastic and immunomodulating agents	6	5.0
L01A Alkylating agents (Temozolomide)	1	0.8
L03 Immunostimulants (Interferon alfa-2b)	3	2.5
L04 Immunosuppressants (Belimumab)	2	1.7
M. Musculo-skeletal system	24	20.2
M01 Anti-inflammatory and antirheumatic products (Ibuprofen)	22	18.5
M02 Topical products for joint and muscular pain (Benzydamine)	2	1.7
N. Nervous system	43	36.1
N01B Anesthetics, local (Procaine)	1	0.8
N02 Analgesics (Paracetamol)	9	7.6
N03 Antiepileptics	32	26.9
N03AF Carboxamide derivatives (Carbamazepine)	4	3.4
N03AG Fatty acid derivatives (Valproic acid)	5	4.2
N03AX Other antiepileptics	23	19.3
Lamotrigine	19	16.0
Levetiracetam	4	3.4
N06 Psychoanaleptics	1	0.8
N06BX Other psychostimulants and nootropics (bovine cerebral cortex polypeptides)	1	0.8
R Respiratory system	9	7.6
R05 Cough and cold preparations		
Butamirate	1	0.8
R06 Antihistamines for systemic use	8	6.7
R06AB Substituted alkylamines (Dimetindene)	1	0.8
R06AD Phenothiazine derivatives (Alimemazine)	1	0.8
R06AE Piperazine derivatives (Cetirizine)	5	4.2
R06AX Other antihistamines for systemic use (Loratadine)	1	0.8
S Sensory organs	3	2.5
S02 Otologicals (Lidocaine + phenazone)	1	0.8

**Table 8:** Drugs involved in SJS and TEN in the elderly.

Drugs	N (Total—48)	%
A. Alimentary tract and metabolism	2	4.2
A04A Antiemetics and Antinauseants (Granisetron)	1	2.1
A11G Ascorbic acid (Vitamin C), including combinations	1	2.1
B Blood and blood-forming organs	1	2.1
B05BA Solutions for parenteral nutrition (Dextrose)	1	2.1
C. Cardiovascular system	2	4.2
C03C High-ceiling diuretics (Furosemide)	1	2.1
C09 Agents acting on the renin–angiotensin system (Perindopril)	1	2.1
J. Anti-infectives for systemic use	19	39.6
J01 Antibacterials for systemic use	17	35.4
J01A Tetracyclines (Tigecycline)	1	2.1
J01C Beta-lactam antibiotics, penicillins (Ampicillin + sulbactam)	1	2.1
J01D Other beta-lactam antibiotics	8	16.7
J01DB First-generation cephalosporins (Cefazolin)	1	2.1
J01DD Third-generation cephalosporins (Ceftriaxone)	4	8.3
J01DH Carbapenems (Meropenem)	3	6.3
J01FA Macrolides (Azithromycin)	2	4.2
J01MA Fluoroquinolones	3	6.3
Ciprofloxacin	1	2.1
Levofloxacin	1	2.1
Moxifloxacin	1	2.1
J01XA Glycopeptide antibacterials (Vancomycin)	2	4.2
J02 Antimycotics for systemic use (Voriconazole)	1	2.1
J07B Viral vaccines	1	2.1
L. Antineoplastic and immunomodulating agents	10	20.8
L01A Alkylating agents (Ifosfamide)	1	2.1
L01B Antimetabolites	2	4.2
Gemcitabine	1	2.1
Capecitabine	1	2.1
L01D Cytotoxic antibiotics and related substances (Doxorubicin)	1	2.1
L01E Protein kinase inhibitors	3	6.3
Ibrutinib	2	4.2
Afatinib	1	2.1
L01F Monoclonal antibodies and antibody–drug conjugates (Pembrolizumab)	1	2.1
L02B Hormone antagonists and related agents	2	4.2
Enzalutamide	1	2.1
Apalutamide	1	2.1
M. Musculo-skeletal system	5	10.4
M01 Anti-inflammatory and antirheumatic products	3	6.3
M01A—Anti-inflammatory and antirheumatic products, non-steroids	3	6.3
Tenoxicam	1	2.1
Meloxicam	1	2.1
Ibuprofen	1	2.1
M02 Topical products for joint and muscular pain	1	2.1
M02AA Anti-inflammatory preparations, non-steroids for topical use (Ketoprofen)	2	4.2
N. Nervous system	8	16.7
N02 Analgesics	2	4.2
N02BA Salicylic acid and derivatives (Acetylsalicylic acid)	1	2.1
N02BE Anilides (Paracetamol)	1	2.1
N03 Antiepileptics	4	8.3
N03AF Carboxamide derivatives (Carbamazepine)	1	2.1
N03AX Other antiepileptics (Lamotrigine)	3	6.3
N05 Psycholeptics	2	4.2
N05AD Butyrophenone derivatives (Droperidol)	1	2.1
N05BX Other anxiolytics (Phenazepam)	1	2.1
S Sensory organs	1	2.1
S01 Ophthalmologicals (Levofloxacin)	1	2.1

## Data Availability

Data were gained from https://newmimn.roszdravnadzor.gov.ru/, accessed on 20 January 2024.
